# Clinically Isolated IgG4-Related Disease of the Larynx: A Rare Cause of Progressive Dysphonia

**DOI:** 10.7759/cureus.100060

**Published:** 2025-12-25

**Authors:** Hunter W Brady, Jasneet Gill, Kaneez Leonard-Bowden

**Affiliations:** 1 Medicine, Lincoln Memorial University DeBusk College of Osteopathic Medicine, Knoxville, USA; 2 Internal Medicine, Tennova North Knoxville, Knoxville, USA; 3 Family Medicine, Tennova North Knoxville, Knoxville, USA

**Keywords:** airway obstruction, dysphonia, igg4 related disease, laryngeal fibrosis, laryngology, plasma cell infiltrates, storiform fibrosis, supraglottic mass

## Abstract

Immunoglobulin G4 (IgG4)-related disease (IgG4-RD) is a systemic immune-mediated fibroinflammatory condition marked by lesions with dense IgG4-positive plasma cell infiltrates. While it is commonly associated with salivary and pancreatic involvement, isolated laryngeal involvement is extremely rare, with an estimated 15 documented cases. This report describes the case of a 66-year-old female presenting with persistent dysphonia who was found to have isolated IgG4-RD of the larynx, highlighting the diagnostic complexity and novel presentation.

The patient presented to the primary care office with persistent and progressive dysphonia following an upper respiratory infection. Laryngoscopy demonstrated a supraglottic submucosal mass. Biopsy showed chronic inflammation, fibrosis, and squamous metaplasia without dysplasia. She was later referred to a tertiary facility for further investigation due to the progressive nature of her symptoms and concern for airway compromise. At that time, the prior biopsy specimen underwent immunohistochemical review for the first time. Immunohistochemical analysis revealed dense lymphoplasmacytic infiltration, storiform fibrosis, and abundant IgG4-positive plasma cells, establishing the diagnosis of IgG4-RD of the larynx. She was initiated on systemic corticosteroids and transitioned to long-term azathioprine, resulting in symptomatic improvement and regression of the laryngeal findings.

IgG4-RD of the larynx is a rare manifestation that can be challenging to diagnose. This report emphasizes the importance of immunohistochemical evaluation in patients whose biopsy results are nondiagnostic. Early recognition of IgG4-RD enables timely treatment, reduces the risk of airway compromise, and helps preserve laryngeal function.

## Introduction

Dysphonia, defined as a change in voice quality, is a common otolaryngologic complaint. Etiologies range from benign conditions, such as acute laryngitis, to more serious causes, including malignancy [1]. While acute laryngitis remains the most prevalent cause, less common etiologies, such as amyloidosis, sarcoidosis, and other granulomatous diseases, may also involve the larynx [1]. Immunoglobulin G4-related disease (IgG4-RD) is a systemic immune-mediated fibroinflammatory disorder characterized by IgG4-positive plasma cell infiltration and fibrosis [2,3]. Although the disease can affect multiple organs, including the pancreas, salivary glands, biliary tract, and kidneys, laryngeal involvement is exceedingly uncommon, with fewer than twenty reported cases [4,5]. Symptoms frequently overlap with more common laryngeal conditions, including chronic laryngitis, idiopathic laryngotracheal stenosis, and neoplasms, contributing to misdiagnosis or delayed recognition [4,5].

Laryngeal involvement may manifest with progressive dysphonia, dyspnea, stridor, or airway obstruction. Histopathologic features classically include IgG4-rich lymphoplasmacytic infiltration, storiform fibrosis, and obliterative phlebitis. Lesions typically respond to glucocorticoids, while steroid-sparing agents such as rituximab may be used for relapse prevention [6,7]. This report discusses the case of a 66-year-old female presenting with progressive dysphonia, who was ultimately diagnosed with isolated laryngeal IgG4-RD, highlighting diagnostic challenges and response to immunosuppressive therapy.

## Case presentation

A 66-year-old female with a past medical history of gastroesophageal reflux disease, type 2 diabetes mellitus, and hepatitis B core antibody positivity presented to her primary care physician with acute-onset, progressive dysphonia following an upper respiratory tract infection. She reported that her voice had abruptly become a whisper and did not recover despite multiple courses of antibiotics and corticosteroids. In addition to progressive dysphonia, the patient reported unintentional weight loss, fatigue, dysphagia to both solids and liquids, frequent aspiration events, nocturnal cough, dyspnea, and stridor at rest.

**Figure 1:**
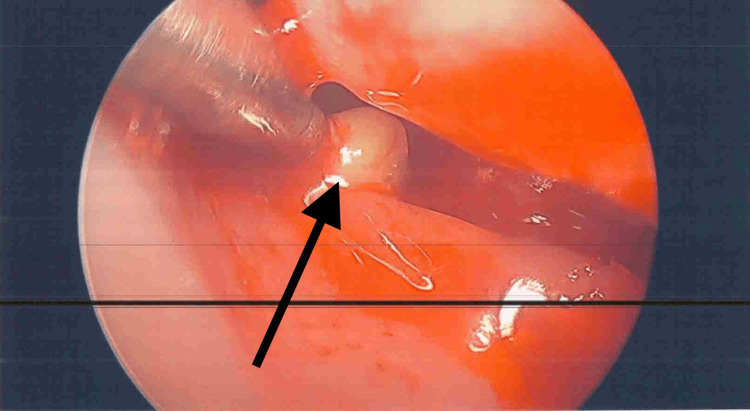
Initial laryngeal examination findings Initial laryngeal examination showing diffuse edema, polypoid changes, and a prominent supraglottic submucosal lesion (black arrow) contributing to impaired visualization of the true vocal cords

Initial laryngoscopic examinations revealed diffuse edema, polypoid changes, and impaired visualization of the true vocal cords (Figure 1). Initial serial biopsies demonstrated chronic inflammation, fibrosis, and squamous metaplasia without dysplasia. Given the persistence and severity of her symptoms, she was referred for tertiary care laryngology evaluation at a large academic medical center. She underwent videostroboscopy, which revealed a large, erythematous, polypoid lesion involving the ventricular folds and anterior commissure and the inability to clearly visualize the true vocal cords.

Laboratory evaluation, including inflammatory markers, autoimmune serologies, immunoglobulin levels, complete blood count, and renal function testing, did not reveal disease-specific abnormalities and failed to provide additional diagnostic insight (Table 1). Further immunohistochemistry re-review of a prior biopsy demonstrated abundant IgG4-positive plasma cells (>30 per high-power field) and an IgG4/IgG ratio of 60%, clinically isolated IgG4-RD of the larynx. Rheumatology consultation confirmed the diagnosis of IgG4-RD with high disease burden. No clinical, laboratory, or imaging findings suggested any involvement of other commonly affected organs, including the pancreas, kidneys, or mediastinum, at the time of diagnosis.

**Table 1:** Summary of laboratory evaluation

Laboratory study	Result	Reference range
Erythrocyte sedimentation rate (ESR)	55 mm/hr	2–37 mm/hr
C-reactive protein (CRP)	2.1 mg/L	0.0–5.0 mg/L
Antinuclear antibody (ANA)	Negative	Negative
Rheumatoid factor (RF)	<10 IU/mL	0–14 IU/mL
Antineutrophil cytoplasmic antibody (ANCA)	Negative	Negative
Anti-meloperoxidase (MPO)	3.00 units	<20 units
Anti-protease 3 (PR3)	3.00 units	<20 units
Immunoglobulin G4 (IgG4)	57 mg/dL	1–123 mg/dL
Angiotensin-converting enzyme (ACE)	27 U/L	9–67 U/L
Hemoglobin	11.9 g/dL	11.8–16.0 g/dL
White blood cells	9.2 ×10³/µL	3.9–10.7 ×10³/µL
Platelets	297 ×10³/µL	135–371 ×10³/µL
Blood urea nitrogen (BUN)	14 mg/dL	8–26 mg/dL
Creatinine	0.79 mg/dL	0.57–1.11 mg/dL

Based on this diagnosis, the patient was initiated on prednisone 20 mg daily, followed by azathioprine 125 mg daily, with close monitoring of her diabetes and hepatitis B core antibody positivity. On follow-up several months later, she demonstrated improvement in vocal strength and laryngeal examination findings without any major adverse effects.

## Discussion

This report describes a patient who developed acute-onset dysphonia following an upper respiratory tract infection that progressed to significant upper aerodigestive dysfunction, highlighting the diagnostic complexity of IgG4-RD of the larynx. The patient’s persistent dysphonia following an upper respiratory tract infection was initially attributed to infectious or inflammatory laryngitis. However, potential differentials included laryngeal changes due to chronic gastroesophageal reflux disease and malignancy. Due to her worsening symptoms and failure to improve, serial laryngeal investigations, biopsies, and laboratory testing were performed, demonstrating nonspecific chronic inflammation, fibrosis of the larynx, and no disease-specific laboratory abnormalities. Such findings contributed to a prolonged diagnostic delay, underscoring the potential limitations of relying on serologic studies. 

Although elevated serum IgG4 levels can support the diagnosis of IgG4-RD, normal levels do not rule out the condition, as demonstrated in this case [8]. Our patient's serum IgG4 results were within reference range despite diagnostic immunohistochemical findings, highlighting the need for immunohistochemical review even when pathology findings are nondiagnostic. Notably, the patient’s erythrocyte sedimentation rate was moderately elevated; however, the C-reactive protein was within normal limits, and complete blood count parameters demonstrated only mild anemia without leukocytosis, suggesting that the ESR elevation was nonspecific, potentially influenced by non-inflammatory factors, and offered little in terms of diagnostic direction.

The rarity of clinically isolated laryngeal involvement in IgG4-RD poses additional diagnostic challenges. IgG4-RD often affects the pancreas, biliary tract, and salivary glands; however, involvement of the upper airway is unusual, leading to prolonged misdiagnosis as more common laryngeal conditions. Reported laryngeal manifestations include supraglottic fullness, subglottic stenosis, vocal fold immobility, and diffuse laryngeal edema [9-11]. The location of the involvement may dictate symptom severity, and in advanced cases, can result in acute airway compromise [9,11]. In the case described here, progressive dysphonia, stridor, and dysphagia signaled significant airway involvement and disease progression.

Most patients with IgG4-RD demonstrate improvement with glucocorticoids, which currently remain the first-line treatment. Among patients treated with glucocorticoids, relapse is common, necessitating steroid-sparing agents such as azathioprine, mycophenolate, or rituximab [12]. Rituximab, a monoclonal anti-CD20 antibody, has shown particular promise in relapsing disease due to its B-cell-directed mechanism of action [13]. This patient responded well to moderate-dose corticosteroids followed by azathioprine maintenance with improved laryngeal findings and symptomatic relief over time. The patient's hepatitis B core antibody further complicated management and required careful monitoring given the risk for viral reactivation with immunosuppressive therapy, an important consideration for long-term treatment strategy.

This report adds to a limited but growing body of literature describing IgG4-RD, specifically of the larynx. Early recognition remains essential to preserve laryngeal function as untreated IgG4-RD can lead to irreversible fibrosis and impairment [14]. This case illustrates that diagnostic confirmation often requires a multidisciplinary approach, involving laryngology, pathology, and rheumatology. Heightened awareness among otolaryngologists is critical, as IgG4-RD should be considered in cases of unexplained progressive dysphonia or atypical inflammatory lesions that are unresponsive to standard therapy. Timely biopsy with immunohistochemical staining can shorten the time to diagnosis and improve patient outcomes.

## Conclusions

Laryngeal IgG4-RD is a rare and underrecognized cause of progressive dysphonia. This report highlights the key challenges in its diagnosis and the importance of immunohistochemical confirmation, especially in the setting of normal serologic markers and nondiagnostic biopsy findings. Prompt treatment with glucocorticoids and steroid-sparing agents can lead to significant symptomatic improvement and regression of laryngeal pathology. Continued reporting of laryngeal involvement is needed to better define the disease spectrum and guide evidence-based management.
